# Brain-resident myeloid cells promote rapid leukocyte adhesion in leptomeningeal vessels after anti-Aβ immunotherapy

**DOI:** 10.64898/2026.09.07.749938

**Published:** 2026-09-11

**Authors:** Moustafa Algamal, Shinya Yokomizo, Wadzanai Ndambakuwa, Sarena Abdallah, Maria V. Sanchez-Mico, Rebecca Gillani, Patrick da Silva, Aryan Rajput, Lakshita Palanivelu, Yuna Lee, Katy R. Walsh, Sunny Kumar, Kivilcim Kilic, Joseph El Khoury, Saef Izzy, Mariel G. Kozberg, Brian J. Bacskai

## Abstract

Anti-amyloid-β (Aβ) immunotherapy improves cognitive outcomes in Alzheimer’s disease (AD) but is associated with amyloid-related imaging abnormalities (ARIA), through poorly understood mechanisms. To define how anti-Aβ antibodies acutely engage brain immune and vascular compartments, we developed a longitudinal in vivo two-photon imaging platform to track microglial dynamics, peripheral immune cell recruitment, and vascular responses in APP/PS1 mice. Anti-Aβ antibodies, including aducanumab and lecanemab, rapidly initiated microglial activation and spatial reorganization within 24 hours of dosing, with recruitment to plaque-associated regions, stabilizing plaque growth. Aducanumab and lecanemab also triggered a rapid and transient cerebrovascular immune response characterized by rolling and adhesion of peripheral immune cells along leptomeningeal vessels, accompanied by endothelial activation. Immune cell characterization revealed recruitment of innate immune cells (Iba1⁺Ki67⁺ monocytes and Ly6G⁺ neutrophils) and proliferative CD3⁺ T cells into the vascular compartment following treatment. Prophylactic treatment with high-dose dexamethasone reduced the number of adherent cells without affecting microglial activation. Depletion of brain-resident immune cells similarly reduced peripheral immune cell recruitment, supporting their contribution to leukocyte recruitment. Postmortem brain tissue from an AD patient treated with lecanemab showed higher proliferation-associated monocyte signature scores, suggesting translational relevance to the proliferative myeloid response observed in our mouse models. These findings demonstrate that anti-Aβ immunotherapy rapidly initiates a coordinated central and peripheral immune response at the leptomeningeal interface. These early immune–vascular interactions represent a plausible initiating mechanism for ARIA and provide a mechanistic framework to guide strategies for mitigating ARIA risk.

## Full Text Availability

The license terms selected by the author(s) for this preprint version do not permit archiving in PMC. The full text is available from the preprint server.

